# Case Report: A female patient with gastrointestinal perforation, acute diffuse peritonitis, and Crohn's disease needs homeostasis management

**DOI:** 10.3389/fmed.2025.1557305

**Published:** 2025-07-29

**Authors:** Dai-Liang Fei, Ze Yu, Wan-Neng Yan, Jin-Liang Dong

**Affiliations:** ^1^Department of General Surgery, Zhoushan Hospital, Wenzhou Medical University, Zhoushan, China; ^2^Department of Anorectum, Zhoushan Hospital, Wenzhou Medical University, Zhoushan, China

**Keywords:** gastrointestinal perforation, acute diffuse peritonitis, Crohn's disease, homeostasis management, acute renal failure

## Abstract

A 47-year-old married woman presented with abdominal discomfort, distension, and nausea following a two-day episode of constipation accompanied by reduced urine output. Upon her admission, a physical assessment indicated tachycardia and abdominal sensitivity, while imaging examinations corroborated gastrointestinal perforation along with signs of acute diffuse peritonitis. The patient had a prior history of Crohn's disease and had been off medication for over a year. The initial surgical intervention entailed bowel resection and adhesiolysis; however, postoperative complications arose, prompting a second operation due to suspected anastomotic leakage and a severe infection. In spite of these challenges, the patient received intensive care, nutritional support, and treatment for short bowel syndrome. Over span of a month, her health significantly improved, and she was ultimately discharged with ongoing management for her Crohn's disease. This case underscores the intricacies involved in addressing intestinal perforations in patients with pre-existing Crohn's disease and highlights the imperative for prompt surgical action and comprehensive postoperative care.

## 1 Introduction

Crohn's disease (CD) is a long-lasting inflammatory condition of the bowel that can cause inflammation throughout the entire gastrointestinal tract, but it most commonly affects the ileum and colon. Individuals with CD frequently experience a range of symptoms such as abdominal pain, diarrhea, weight loss, and fatigue, all of which can greatly diminish their quality of life. To diagnose CD, healthcare providers usually rely on a mix of clinical assessments, imaging tests, and endoscopic examinations, which often show signs like strictures, ulcers, or fistulas in the inflamed sections of bowel (1). The management of CD often includes pharmacological therapies designed to induce and maintain remission. However, in certain situations, surgical intervention becomes essential, particularly when complications arise, such as bowel obstruction, perforation, or when the disease does not respond adequately to medical treatment (2).

The case discussed involves a 47-year-old woman with a history of Crohn's disease who experienced gastrointestinal perforation, which resulted in acute diffuse peritonitis and subsequent septic shock. This situation is uncommon and presents considerable clinical challenges, especially in patients with pre-existing inflammatory bowel disease. The significance of this case is its potential to inform differential diagnoses for patients who present with acute abdominal symptoms, particularly those with a known diagnosis of Crohn's disease. Managing such cases necessitates a collaborative approach that includes prompt surgical intervention and thorough postoperative care to reduce complications and enhance patient outcomes.

## 2 Case presentation

### 2.1 Clinical presentation

The patient presented with abdominal pain and distension for 2 days, accompanied by sensations of limb spasms. She reported nausea and vomiting the following day, expelling a small amount of liquid. Over the past 2 days, she experienced cessation of flatus and bowel movements, with worsening abdominal pain. The patient had minimal oral intake, restricted to a small amount of water, and noted a significant decrease in urine output. Subsequently, she was transported to the emergency department.

### 2.2 Admission examination

- **Vital signs:**

Temperature: 36.7°C; Pulse: 151 beats per minute;

Respiration: 18 breaths per minute; Blood Pressure:

106/64 mmHg; Consciousness: alert.

- **Physical examination:**

Skin and sclera: no jaundice, edema, or rash;

Chest: symmetrical thorax, clear breath sounds bilaterally;

Heart: regular rhythm, strong heart sounds.

- **Abdomen:** distended, with generalized tenderness, rebound tenderness, and rigidity; negative shifting dullness; tenderness in the liver area.
- **Extremities:** no deformities, tenderness, or joint limitations.

### 2.3 Auxiliary examinations

**Figure 1:**
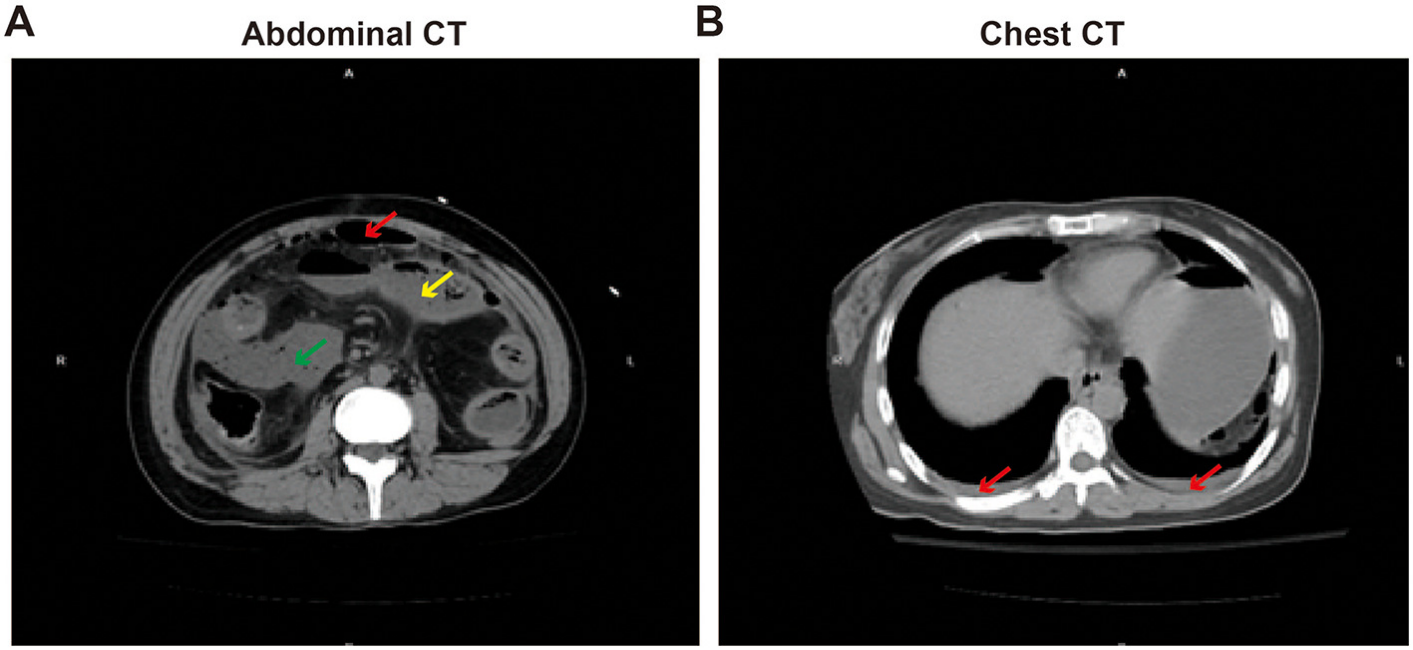
Abdominal and chest CT imaging diagnosis. **(A)** Abdominal CT, The red arrow mark free gas in the abdominal cavity and perforation of the gastrointestinal tract; yellow arrow indicates mild intestinal distension and fluid presence; the green arrow indicates the presence of peritoneal effusion. **(B)** Chest CT, the red arrow indicates a small amount of pleural effusion.

**Imaging:** the CT scan of the abdomen and chest revealed gastrointestinal perforation, with portions of the bowel slightly distended and presence of fluid. There was a small amount of fluid in the abdominal and pelvic cavities, as well as minimal effusion in both pleural cavities (Figure 1).

### 2.4 Medical history

The patient has a 10-year history of Crohn's disease, currently in remission as she has been off medication for 1 year.

### 2.5 Preliminary diagnosis

Gastrointestinal perforation; Acute diffuse peritonitis; Septic shock; Acute renal failure and Crohn's disease.

### 2.6 Surgical record 1

Under combined the intravenous and inhalation anesthesia, the patient underwent bowel resection and adhesion lysis. After successfully inducing general anesthesia, the patient was positioned supine, and the surgical field was disinfected as per routine protocols. A midline incision, approximately 25 cm in length, was made, which revealed around 500 ml of feculent fluid in the abdominal cavity. Multiple dense adhesions were noted among the lower abdominal bowel, peritoneum, and mesentery. Adhesions were carefully separated using an electrocautery device, during which significant edema and thickening of the omentum, small intestine, and colon were observed, accompanied by purulent exudate and fecal matter. A perforation, roughly 1 cm in diameter, was identified in the mesenteric border of the ileum, located about 60 cm from the cecum, with feculent fluid present at the site. Following extensive irrigation of the abdominal cavity with saline, the adjacent ileal segment showed signs of congestion and edema, with focal necrotic changes noted. Considering the patient's history of Crohn's disease and poor intestinal quality, the mesangial vessels were severed and ligation, and end-to-end anastomosis was performed and the anastomosis was reinforced. The affected segment was resected, and an end-to-end anastomosis was performed using absorbable sutures. After confirming that there was no tension or stricture at the anastomosis site, the mesenteric defect was closed. Two drainage tubes were placed near the perforation site, and suction drains were positioned in the hepatic flexure and the splenic fossa. The procedure concluded with careful closure of all layers and a confirmation of instrument counts.

### 2.7 Postoperative diagnosis

**Figure 2:**
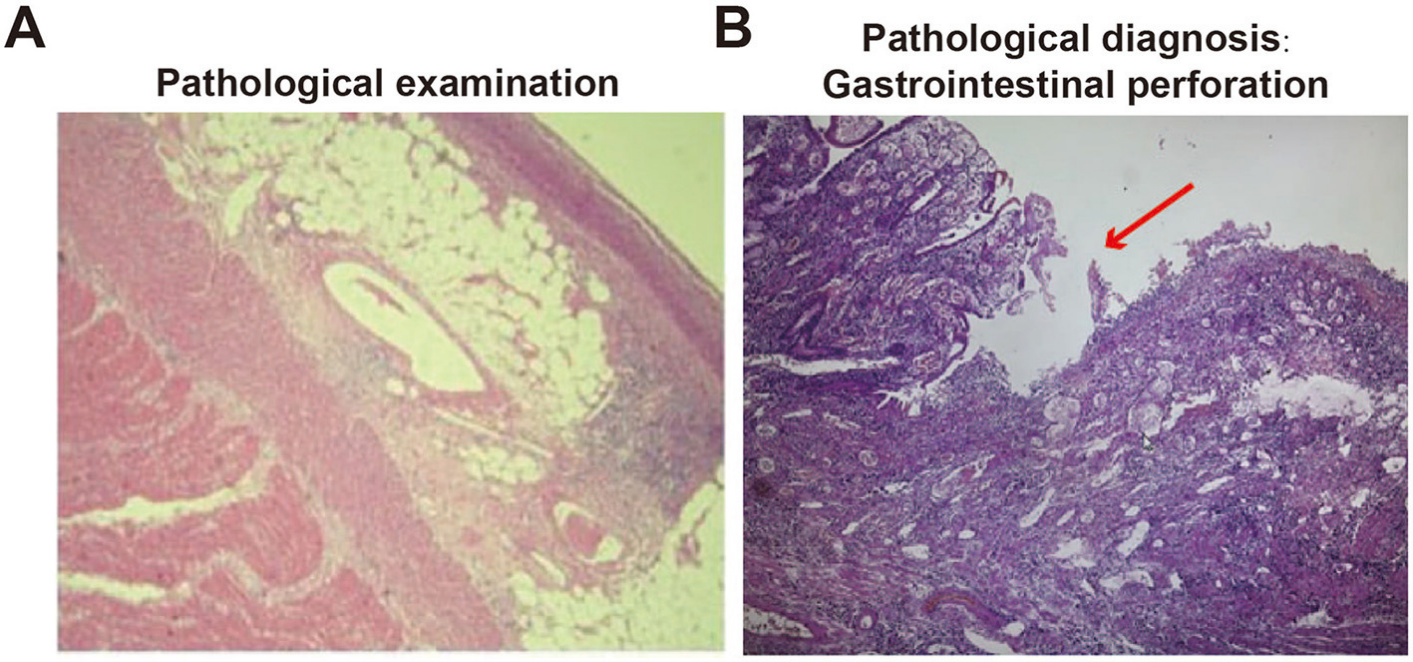
Pathological examination of perforated small intestine. **(A)** Pathological examination of small intestinal perforation; **(B)** gastrointestinal perforation, mucosal loss (pathological diagnosis, red arrow).

- Gastrointestinal perforation (Pathological diagnosis, Figure 2)
- Acute diffuse peritonitis
- Acute renal failure
- Crohn's disease
- Adhesions
- Septic shock

### 2.8 Postoperative recovery

The patient was moved to the Intensive Care Unit (ICU) for close monitoring and specialized care. Throughout this period, vital signs remained stable, and the drainage output was clear and unobstructed. In the subsequent days, the patient began to pass gas and have bowel movements, demonstrating improvement in gastrointestinal function. Additionally, the patient was able to walk with assistance and gradually transitioned to a liquid diet, with notable progress in the recovery observed.

However, the patient later developed acute facial expression changes, dyspnea, tachycardia (heart rate of 135 bpm), and hypoxemia (oxygen saturation at 93% with a reservoir mask). Abdominal distension with mild tenderness was noted, prompting an ICU transfer for continued management. The patient required intubation and mechanical ventilation support. She was in a sedated state with systemic signs of infection, and the drainage tube was yielding purulent fluid, including dark green liquid. Given her history of Crohn's disease and ongoing immunosuppression, severe postoperative infection was suspected, as well as possible anastomotic leakage or recurrent bowel perforation, necessitating emergency exploratory laparotomy.

## 3 Surgical record 2

Following successful general anesthesia, a re-exploration was conducted through the original midline incision. Upon entering the abdominal cavity, a mass consisting of omentum, bowel, and associated mesentery was discovered, all covered in a significant amount of purulent exudate. There was also an accumulation of feculent fluid surrounding the previous anastomosis, which extended to adjacent areas. Dense adhesions were observed between the lower abdominal bowel, peritoneum, and mesentery, accompanied by notable edema. A perforation, approximately 0.5 cm in diameter, was identified near the mesenteric border of the ileum, located 10 cm from the cecum, with feculent fluid present. After thoroughly irrigating the abdominal cavity, the stomach, small intestine, and colon were examined, revealing no further perforations, strictures, tears, or neoplastic lesions. Given the severity of the patient's Crohn's disease and the extent of the infection, the surgical team decided to perform a double-barreled ileostomy. The ileum was transected at the site of the original anastomosis, with the distal segment positioned about 10 cm from the cecum and the proximal segment measuring approximately 90 cm. Due to the limited remaining length of bowel and the high risk of electrolyte imbalance and nutritional deficiency, the double-barreled ileostomy was deemed necessary.

The creation of the ostomy was complicated by significant edema and rigidity in the mesentery. The stoma was established on the right abdominal wall and secured in layers to both the peritoneum and the abdominal wall. The ileal mucosa was well vascularized, and an ostomy bag was subsequently attached. Following extensive irrigation of the abdominal cavity, two drainage tubes were placed adjacent to the stoma, along with suction drains in the hepatic flexure and splenic fossa. The procedure was concluded with careful closure of all incisions.

### 3.1 Postoperative monitoring

The patient was readmitted to the ICU for intensive monitoring due to intestinal obstruction and bowel necrosis, which resulted in acute peritonitis. She was placed on a regimen of combined antibiotic therapy, specifically imipenem-cilastatin and linezolid, and required ventilatory support, sedation, proton pump inhibitors, and electrolyte replenishment. Throughout her treatment, healthcare providers closely monitored her abdominal signs and drainage output, remaining alert for potential complications such as pain, bleeding, and electrolyte imbalances.

After stabilization, the patient was moved to a general ward where they received level I nursing care and continued their antibiotic treatment. Nutritional support was administered through both enteral and parenteral methods, with gradual adjustments made to the somatostatin infusion. Initially, the ostomy output was measured at 2,200 ml of yellow, turbid fluid, which indicated the presence of short bowel syndrome and a significant electrolyte imbalance, leading to the need for intravenous hydration. Nutrition specialists provided recommendations for a combined approach to enteral and parenteral nutritional support. Over time, the ostomy output decreased to about 500 ml per day, and oral potassium chloride supplementation was started to help stabilize the patient's internal environment. Additionally, the patient developed metabolic acidosis, which was addressed with sodium bicarbonate treatment.

The abdominal drainage tube was removed, and a gastroenterology consultation was requested to manage Crohn's disease. Supportive care continued, focusing on monitoring stoma output and vital signs. Over the next month, the infection gradually resolved, leading to the discontinuation of intravenous antibiotics. Nutritional support was maintained; however, the extended use of total parenteral nutrition (TPN) resulted in liver function impairment. This complication required treatment with compound glycyrrhizin to protect the liver and reduce enzyme levels. As the patient's oral intake improved, oral enteral nutrition was increased, and TPN was gradually reduced.

### 3.2 Follow-up and outcome

**Figure 3:**
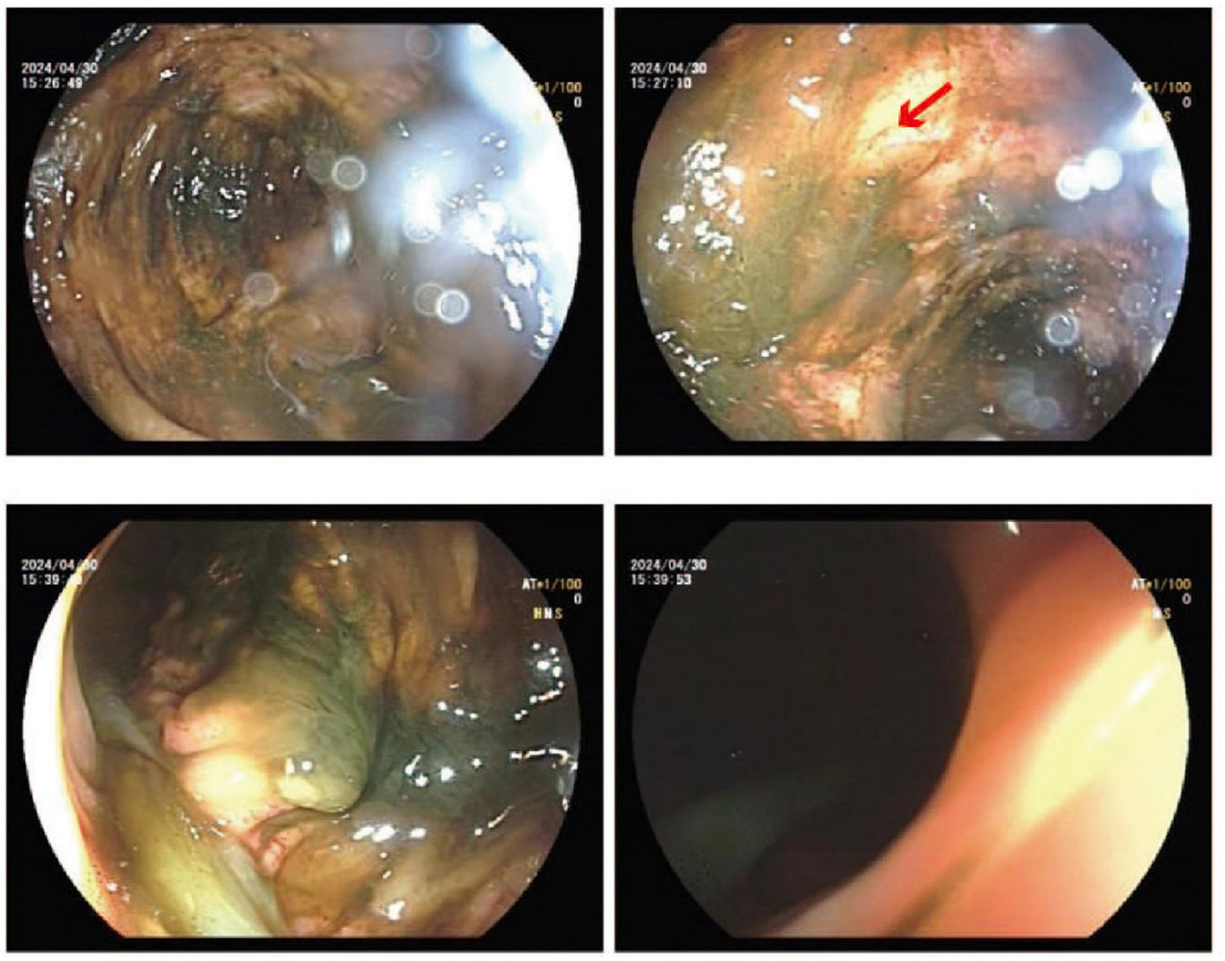
Endoscopic catheterization diagnosis. From the lower ileum to the transverse colon, multiple flaccid erosion and feces can be seen in the colon. The red arrow indicates flaccid erosion.

After a 20-day period, the patient underwent catheter placement through endoscopy, where a tube was inserted from the distal ileostomy to the ascending colon (Figure 3). This procedure aimed to enhance the absorption of water and electrolytes, ultimately improving the patient's electrolyte balance. Following the intervention, the patient started to experience watery stools from the stoma. Family members were advised to administer small amounts of water into the colon while also encouraging the patient to maintain oral nutritional intake.

The patient maintained oral nutrition, small meals, increased intestinal fluid flow, enhanced absorption, and balanced intake and outflow.

One month after her initial treatment, the patient was discharged home with oral nutritional support, along with self-prepared nutritional liquids. During this time, her internal environment stabilized, and she demonstrated remarkable recovery. Six months following her discharge, the patient successfully underwent an ileostomy reversal procedure, which effectively reconstructed her digestive tract.

## 4 Discussion

This case underscores the complexities involved in managing a patient with Crohn's disease who experienced gastrointestinal perforation, resulting in acute diffuse peritonitis. Such cases are particularly concerning due to the significant risk of morbidity and mortality linked to delays in diagnosis and treatment. Previous studies have shown that individuals with a history of Crohn's disease face a heightened risk of gastrointestinal complications, including perforation, which can lead to serious consequences if not addressed swiftly (3).

The patient's postoperative complications, which include infection and the risk of anastomotic leakage, highlight the difficulties associated with surgical procedures in individuals with underlying inflammatory bowel disease. Previous studies reported that postoperative infections occur more frequently in patients with Crohn's disease, primarily due to the impaired healing processes linked to inflammation and malnutrition (4). Besides, the occurrence of anastomotic leaks complicates recovery, often necessitating further surgical interventions and extended hospital stays, as demonstrated by the patient's requirement for multiple surgeries detailed in this report (5).

The choice to perform an enterostomy instead of a primary anastomosis is a widely accepted surgical strategy when dealing with compromised intestinal quality, especially in patients with Crohn's disease. Previous studies reported that although an enterostomy can serve as a short-term solution, it brings about significant long-term complications. These complications often include nutritional deficiencies and electrolyte imbalances, particularly in situations where the length of the remaining bowel is inadequate for proper nutrient absorption (6). The utilization of distal enteral feeding, as described in this case, has shown promise in maintaining nutritional status without reliance on parenteral nutrition, which is laden with complications (7).

Long-term management of patients with short bowel syndrome requires a collaborative approach that involves various healthcare professionals. This strategy highlights the critical role of nutritional support, as well as the need for careful monitoring to identify and address potential complications such as electrolyte imbalances and dehydration. By ensuring that patients receive comprehensive care tailored to their unique needs, healthcare teams can significantly improve outcomes and enhance the quality of life for those affected by this condition (8). The case illustrates that incorporating growth factors and dietary changes can greatly improve recovery and overall quality of life. Nonetheless, it is crucial to acknowledge the heightened risk of recurrent infections and the necessity for ongoing monitoring, particularly in light of the patient's underlying condition of Crohn's disease. This chronic illness demands continuous management to prevent exacerbations and potential complications (4).

In addition, we also found a similar case report that discusses acute peritonitis caused by intestinal perforation in patients with Crohn's disease, emphasizing potential serious complications of the disease and the necessity of surgical intervention (9). The patients in these articles presented with abdominal pain and gastrointestinal symptoms, and were diagnosed with diffuse peritonitis caused by terminal ileal perforation upon investigation, treated through intestinal resection (such as anastomosis or stoma), which aligns with common strategies in the management of Crohn's disease. However, the key differences lie in the disease history and clinical outcomes: the patient in our case had a clear history of Crohn's disease but had not taken medication for over a year, leading to specific prodromal symptoms such as constipation and reduced urine output before perforation, and required a second surgery and short-term nutritional support treatment postoperatively due to complications like anastomotic leakage to address short bowel syndrome, ultimately achieving improvement and discharge under multidisciplinary care; in contrast, the patient reported in the other case had only a one-year history of symptoms, the surgery was smooth with no complications, and there was a lack of details on postoperative management, with the discussion leaning more towards pathological and technical considerations.

The advantage of our case report lies in its comprehensiveness and clinical practicality: it not only presents the dynamic management process from the initial onset of symptoms to final recovery (including initial misjudgment, second surgery, intensive care, nutritional intervention), but also highlights complexity of Crohn's disease perforation (such as the impact of medication interruption) and the importance of comprehensive postoperative care (such as management of short bowel syndrome), providing a valuable example for diagnosis, treatment, and long-term management of similar complex cases.

## 5 Conclusion

In conclusion, this case highlights the complex relationship between surgical procedures and the management of chronic gastrointestinal issues. It underscores the importance of thorough preoperative evaluations, taking into account the patient's medical history, and establishing detailed postoperative care plans. Moving forward, clinical practices should aim to improve methods for managing patients with Crohn's disease and related conditions to achieve better health outcomes and improve their quality of life. Additionally, there is a need for further research to investigate new nutritional strategies and rehabilitation methods that can help reduce the negative impacts of surgical treatments on this sensitive group of patients.

## Data Availability

The original contributions presented in the study are included in the article/supplementary material, further inquiries can be directed to the corresponding authors.
